# Cyr61 as mediator of Src signaling in triple negative breast cancer cells

**DOI:** 10.18632/oncotarget.3760

**Published:** 2015-04-20

**Authors:** María Pilar Sánchez-Bailón, Annarica Calcabrini, Víctor Mayoral-Varo, Agnese Molinari, Kay-Uwe Wagner, Jesús Pérez Losada, Sergio Ciordia, Juan Pablo Albar, Jorge Martín-Pérez

**Affiliations:** ^1^ Departamento de Biología del Cáncer, Instituto de Investigaciones Biomédicas A. Sols (CSIC/UAM), Madrid 28029, Spain; ^2^ Dipartimento Tecnologie e Salute, Istituto Superiore di Sanità, Roma 00161, Italy; ^3^ Eppley Institute for Research in Cancer and Allied Diseases, University of Nebraska Medical Center, Omaha, NE 68198-6805, USA; ^4^ Centro de Investigación del Cáncer (CSIC/USAL), Campus Unamuno, Salamanca 37007, Spain; ^5^ Servicio de Proteómica, Centro Nacional de Biotecnología (CSIC), Madrid 28049, Spain

**Keywords:** c-Src, Cyr61, triple negative breast cancer, migration, invasion

## Abstract

SFKs are involved in tumorigenesis and metastasis. Here we analyzed c-Src contribution to initial steps of metastasis by tetracycline-dependent expression of a specific shRNA-c-Src, which suppressed c-Src mRNA and protein levels in metastatic MDA-MB-231 cells. c-Src suppression did not alter cell proliferation or survival, but it significantly reduced anchorage-independent growth. Concomitantly with diminished tyrosine-phosphorylation/activation of Fak, caveolin-1, paxillin and p130CAS, c-Src depletion also inhibited cellular migration, invasion and transendothelial migration. Quantitative proteomic analyses of the secretome showed that Cyr61 levels, which were detected in the exosomal fraction, were diminished upon shRNA-c-Src expression. In contrast, Cyr61 expression was unaltered inside cells. Cyr61 partially colocalized with cis-Golgi gp74 marker and with exosomal marker CD63, but c-Src depletion did not alter their cellular distribution. In SUM159PT cells, transient c-Src suppression also reduced secreted exosomal Cyr61 levels. Furthermore, conditional expression of a c-Src dominant negative mutant (SrcDN, c-Src-K295M/Y527F) in MDA-MB-231 and in SUM159PT diminished secreted Cyr61 as well. Cyr61 transient suppression in MDA-MB-231 inhibited invasion and transendothelial migration. Finally, in both MDA-MB-231 and SUM159PT, a neutralizing Cyr61 antibody restrained migration. Collectively, these results suggest that c-Src regulates secreted proteins, including the exosomal Cyr61, which are involved in modulating the metastatic potential of triple negative breast cancer cells.

## INTRODUCTION

The members of Src family of tyrosine kinases (SFKs) are important intracellular mediators of growth factors, cytokines, steroid hormones, etc. [1–8]. Also integrins stimulate the Fak/Src complex, which in turn regulates adhesion, migration, invasion, etc. [9]. Consequently, SFKs control signal transduction pathways that regulate cell division, motility, adhesion, migration, angiogenesis, survival, and differentiation [1, 10–12]. Although there are not consistent evidences for c-Src mutations in human tumors [13, 14], deregulation of SFKs expression and/or activity is associated with tumorigenesis and metastasis [10, 15–17]. Therefore, SFKs are therapeutic targets to treat cancer [18].

Numerous evidences support the role of c-Src in breast cancer. c-Src is overexpressed and/or hyper-activated in breast carcinoma tissue of human biopsies [19, 20]. Additionally, the relevance of Src in mammary tumorigenesis was demonstrated in mice [21]. Overexpression and co-association of Her2/Neu and c-Src have been described in human breast cancer cell lines and tumor samples [15, 22]. Increased expression of Src in tumor cells induces phosphorylation and inactivation of PTEN, facilitating AKT activation and cell survival [23, 24]. Src also promotes proliferation by phosphorylation-induced degradation of p27^kip1^ [25]. Similarly, Src phosphorylates estrogen receptor alpha in human breast cancer and promotes its proteolysis with subsequent therapeutic implications [26].

Src is involved in regulation of migration and invasiveness by controlling focal adhesion turnover, required for breast cancer metastasis [27–31]. In cancer biopsies and metastatic cell lines of human breast, colon and rectal cancer, Src tyrosine phosphorylates caveolin-1 (Y14) increasing tumor cell migration and invasion [32]. The catalytic activity of SFKs is necessary for anchorage-independent growth of metastatic human breast cells [30, 33]. Cancer cells secrete soluble molecules and microvesicles that contribute to cellular communication and cancer dissemination [34]. A comparative analysis of exosomal vesicles composition between no-metastatic MCF7 and MDA-MB-231 breast cancer cells showed, among others, increased levels of matrix metalloproteinases in MDA-MB-231 cells that could be related to their metastatic nature [35]. Furthermore, Src is also involved in angiogenesis and transendothelial migration of different tumor cells [36–39].

Breast cancer is a leading cause of death for women mainly due to metastasis [40] and SFKs are involved in these processes [41–46]. Src kinase activity is required for MDA-MB-231 cells to metastasize to bone [47] lung [48] and brain [49]. Here we analyzed the role of c-Src in metastatic human breast cancer MDA-MB-231 and SUM159PT cells. Results showed that c-Src is not required for MDA-MB-231 cell proliferation or survival, but it significantly reduced growth in soft agar. Depletion of c-Src significantly inhibited migration, invasiveness, and transendothelial migration. Quantitative proteomic analyses of the secretome showed that the levels of Cyr61 (Cysteine-rich protein 61), which was mainly detected in the exosomal fraction, were diminished upon c-Src knockdown. Similarly, c-Src suppression in SUM159PT reduced the amount of Cyr61 in the secretome. Also, a dominant negative variant of this kinase (SrcDN, c-SrcK295M/Y527F) diminished secreted Cyr61 in both cell lines. Furthermore, transient suppression of Cyr61 in MDA-MB-231 cells decreased invasion and transendothelial migration as well. Consistently, neutralizing Cyr61 antibody inhibited MDA-MB-231 and SUM159PT cell migration. Together, these results suggest that Cyr61 is, at least in part, a c-Src mediator in these triple negative breast cancer cell models.

## RESULTS

### Involvement of c-Src in MDA-MB-231 proliferation and anchorage-independent growth

**Figure 1:**
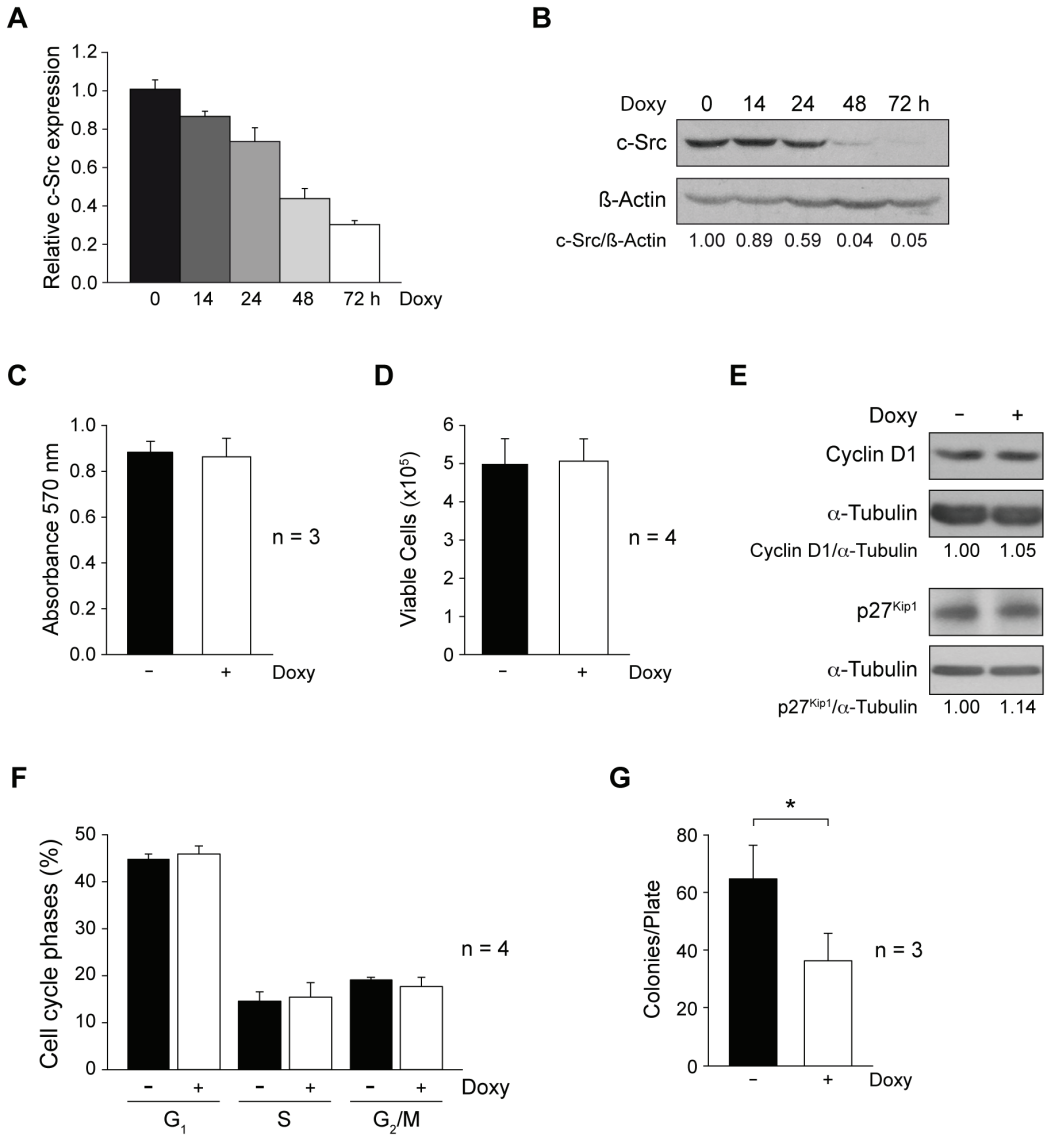
Involvement of c-Src in cell proliferation and anchorage-independent growth of MDA-MB-231-Tet-On-shRNA-c-Src **A.** To analyze c-Src mRNA and protein expression in MDA-MB-231-Tet-On-shRNA-c-Src cells, total RNA and protein were isolated from cells grown with or without Doxy (2 μg/ml) for different times. c-Src mRNA expression was determined by qRT-PCR by a TaqMan assay employing TBP as endogenous control (see Materials and Methods). Results are shown as mean ± SD of relative c-Src mRNA levels in three independent experiments in triplicate, considering arbitrarily the first sample of Doxy-untreated cells triplicate as 1. **B.** Cell extracts were used to detect c-Src by immunoblotting with MAb-327; membranes were then reblotted with anti-β-actin for loading control. Results are representative of three independent experiments. **C.** Cells were grown with or without Doxy (2 μg/ml) for 72 h. Metabolic activity was determined by MTT assay measuring absorbance at 570nm. Results are expressed as mean ± SD from three independent experiments in triplicate. **D.** Cell viability was evaluated counting cells after Trypan blue labeling. Results are shown as mean ± SD from four independent experiments in triplicate. **E.** Extracts from control and Doxy-treated cells were blotted with Cyclin D1 and p27^Kip1^ antibodies. Membranes were reblotted with anti-α-tubulin for loading control. Results are representative of three independent experiments. **F.** Cells were collected fixed with 70% ethanol in PBS at 4°C, washed with PBS and incubated with RNAase and propidium iodide for 1 h at 37°C, cell acquisition was performed with FACScan flow cytometer (BD). Percentages of cell cycle phases (G_1_, S, G_2_/M) were calculated from DNA histograms by CellQuest software. Results are shown as mean ± SD from four independent experiments. **G.** Number of colonies/plate obtained after cell growth in soft-agar (20 days −/+ 2 μg/ml Doxy) (see Material and Methods). Colonies were stained with crystal violet and counted by phase contrast microscopy. Average ± SD from three independent experiments in triplicate (*p < 0.05).

To evaluate whether c-Src suppression altered proliferation and survival of metastatic MDA-MB-231 cells, we first generated a derived cell line with conditional expression (Tet-On) of a specific shRNA for human c-Src, as described in Materials and Methods. Addition of Doxy (Doxycycline, 2 μg/ml) to MDA-MB-231-Tet-On-shRNA-c-Src reduced expression of c-Src mRNA and protein in a time-dependent manner (Figure 1A, B). To confirm the specificity of the shRNA-c-Src, we constitutively expressed chicken c-Src in MDA-MB-231-Tet-On-shRNA-c-Src cells. We observed that depletion of endogenous c-Src upon addition of Doxy was compensated by chicken wild type c-Src expression (Supplementary Figure 1A).

Interestingly, shRNA-c-Src induction did not modify the proliferation of adherent MDA-MB-231-Tet-On-shRNA-c-Src cells. The results from metabolic activity (MTT) and cell viability (Trypan blue) assays (Materials and Methods) were similar in control and Doxy-treated cells (Figure 1C, D). It should be noted that the percentage of Trypan blue-stained cells was always smaller than 5% (data not shown), indicating that c-Src suppression was not cytotoxic. Furthermore, c-Src suppression did not alter expression of cyclin D1 and p27^Kip1^ (Figure 1E). Consistently, flow cytometric analysis of the cell cycle using propidium iodide labeling showed no differences in the percentage of cells in G_1_, S or G_2_/M phases between untreated and Doxy-treated cultures (Figure 1F).

Anchorage-independent growth is a hallmark of malignant-cell transformation. Cells were then cultured in soft-agar in the absence or presence of Doxy and after 20 days, colonies were stained with crystal violet and counted. The results shown in Figure 1G revealed a significant reduction in the number of colonies bigger than 0.1 mm size upon suppression of c-Src. However, the analyses of all colonies (bigger than 20 μm) did not show differences in the number of colonies after c-Src depletion (data not shown). These results suggest that c-Src suppression affected colony cell growth.

### Suppression of c-Src reduced cell migration, transendothelial migration and invasiveness

**Figure 2:**
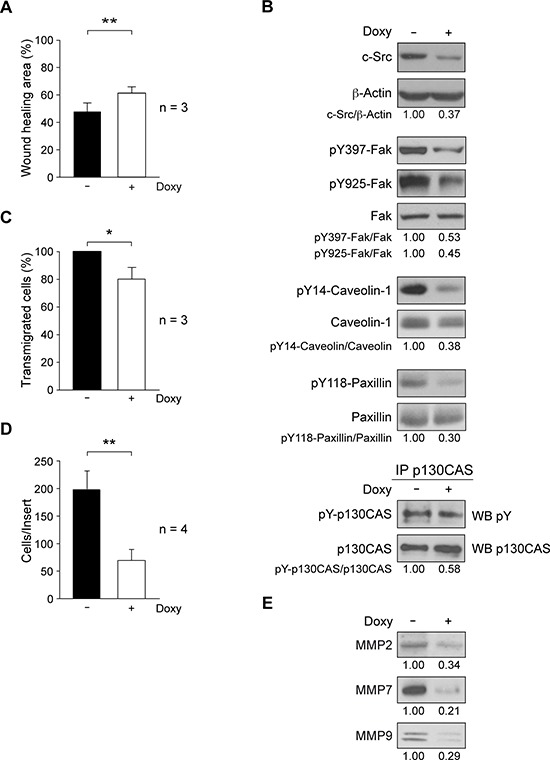
Role of c-Src in migration and invasion properties of MDA-MB-231-Tet-On-shRNA-c-Src cells **A.** Cell migration was determined by wound-healing assay through scratching confluent cultures; photomicrographs were taken at 0 and 20 h with a Microscope Cell Observer Z1 system, and quantified using wound-healing tool of ImageJ. Results are expressed as mean percentage of wound healing area ± SD at 20 h respect to 0 h from three independent experiments (**p < 0.01). **B.** Expression of phosphoproteins/proteins involved in cell motility by immunoblotting. Extracts from control and treated cells (2 μg/ml Doxy, 72 h) were blotted with antibodies to c-Src (MAb-327), pY397-Fak, pY925-Fak, pY14-Caveolin and pY118-Paxillin. p130CAS was immunoprecipitated from total cell extracts and immune-complexes blotted with anti-pY (4G10). Membranes were reblotted with anti-β-actin (for c-Src) and anti-total-protein (for phosphoproteins) for loading control. Results are representative of three independent experiments. **C.** Transendothelial migration through a HUVEC monolayer. Cells were grown for 48 h −/+ 2 μg/ml Doxy and then seeded on the HUVEC monolayer. Transmigrated cells were detached after 22 h and counted in a hemocytometer. The number of Doxy-treated transmigrated cells was expressed as percentage of control transmigrated cells (100%). Assay was repeated three times (*p < 0.05). **D.** For cell invasion through Matrigel-coated inserts, cultures were grown for 48 h −/+ 2 μg/ml Doxy and then seeded onto Matrigel (−/+ 2 μg/ml Doxy); 22 h later, cells on the top of inserts were removed and invaded cells were fixed, stained with DAPI and counted by fluorescence microcopy. The number of invaded cells per insert is shown and represents average ± SD of four experiments in triplicate (**p < 0.01). **E.** Analysis of secreted metalloproteinases MMP2, MMP7 and MMP9 from equal number of control or Doxy-treated cells (2 μg/ml) for 72 h. Conditioned media were used to prepare total soluble fraction of secretome by differential centrifugation (S3, Figure 4A). After concentration by methanol/chloroform precipitation, pellet was resuspended in RIPA for immunoblotting analyses (see Material and Methods). Results are representative of three independent experiments.

We have previously shown that inhibition of Src family tyrosine kinase activity in MDA-MB-231 reduced cell migration [31]. We tested here whether c-Src suppression could modify migration properties. Cells were grown to confluence for 48 h in absence or presence of Doxy (2 μg/ml); after scratching and renewing media −/+ Doxy, cultures were placed in a Microscope Cell Observer and pictures were taken at 0 and 20 h. Analyses of images with the wound-healing tool of ImageJ showed that addition of Doxy to the cultures caused a significant reduction of cell migration (Figure 2A). Furthermore, random migration analysis of sub-confluent cultures showed a significant reduction of the mean velocity and distance travelled by Doxy-treated cells as compared to control (Supplementary Figure 2A, B).

Association of Src with Fak controls turnover of focal adhesion complexes, which are involved in cell motility. Therefore, we analyzed the effect of c-Src suppression on the degree of phosphorylation/activation of Fak, caveolin-1, paxillin and p130^CAS^. Total cell extracts from exponentially growing cultures in absence or presence of Doxy for 72 h were analyzed by immunoblotting. Results showed that shRNA-c-Src induction reduced phosphorylation of Fak at Y397, the autophosphorylation site, and at Y925, which is a substrate for Src tyrosine kinase activity [50]. It also diminished the phosphorylation of caveolin-1 at Y14, paxillin at Y118 and tyrosine phosphorylation of p130^CAS^ (Figure 2B). These results are consistent with the observed decrease in cell migration. The constitutive expression of wild-type chicken c-Src in c-Src depleted cells restored their migration ability. Furthermore, the activation of Fak, caveolin, and paxillin was no longer reduced upon c-Src suppression (Supplementary Figure 1B), confirming the specificity of the shRNA-c-Src employed in our model.

Extravasation and intravasation are events required for tumor-cell metastasis. To test whether c-Src could influence transmigration through endothelium, we performed transendothelial migration assays. Exponentially growing cultures in absence or presence of Doxy for 48 h were seeded on a HUVEC monolayer in cell culture inserts. In parallel, to determine HUVEC spontaneous migration a control was performed in the same conditions by seeding endothelial cells alone. After 22 h, the analysis of transmigrated cells showed that shRNA-c-Src induction by Doxy significantly reduced transendothelial migration (Figure 2C).

Numerous studies support the role of Src kinases in cellular invasion [10]. Since MDA-MB-231 cells are highly metastatic [47], we determined the effect of c-Src suppression in the invasive properties of this cellular model. Control and Doxy-treated cultures (48 h) were seeded onto Matrigel-coated cell culture inserts and allowed to invade for 22 h in the presence or absence of Doxy (see Materials and Methods). The results revealed that c-Src suppression significantly inhibited cellular invasive ability (Figure 2D). In addition, ablation of this kinase reduced the levels of matrix metalloproteinases MMP2, MMP7 and MMP9 in the secretome (Figure 2E). Similar results were obtained in MDA-MB-231-Tet-On-SrcDN that conditionally expresses a dominant negative form of c-Src (SrcDN: c-Src-K295M/Y527F), which is devoid of catalytic activity but with functional SH2 and SH3 domains. Addition of Doxy did not alter cell proliferation but reduced cellular migration and invasion (Supplementary Figure 3A–C). Accordingly, reduced levels of MMP2, MMP7, and MMP9 were identified in the secretome of these cells (Supplementary Figure 3E).

### Proteomic analyses of the secretome

Since c-Src appears to be important for cellular invasiveness, we analyzed whether its ablation modified other secreted proteins in addition to matrix metalloproteinases. Conditioned media were collected from cells grown in absence or presence of Doxy for 72 h. Proteins contained in total secretome (fraction S3, see diagram of Figure 4A) were submitted to quantitative proteomic analyses (HPLC- MS/MS) after labeling tryptic-peptides with iTRAQ. Peptides were then identified employing the two different search engines Mascot and Phenyx (Materials and Methods). The data showed that c-Src suppression diminished the levels of 13 and increased the levels of 7 proteins in secretome (Table 1).

**Table 1:** Quantitative proteomic analysis of secretome from MDA-MB-231-Tet-On-shRNA-c-Src cells

		Mascot AverageRatio Ctrl/Doxy		Phenyx AverageRatio Ctrl/Doxy	
PROTEIN AC	Description	iTRAQ1	iTRAQ2	iTRAQ1	iTRAQ2
O00622	Protein CYR61 OS = Homo sapiens GN = CYR61 PE = 1 SV = 1	3.569	4.423	3.512	3.470
P61769	Beta-2-microglobulin OS = Homo sapiens GN = B2M PE = 1 SV = 1	1.608	1.568	1.575	1.368
P22692	Insulin-like growth factor-binding protein 4 OS = Homo sapiens GN = IGFBP4 PE = 1 SV = 2	1.546	1.415	1.408	1.292
P29279	Connective tissue growth factor OS = Homo sapiens GN = CTGF PE = 1 SV = 2	1.519	1.414	1.541	1.370
P01034	Cystatin-C OS = Homo sapiens GN = CST3 PE = 1 SV = 1	1.441	1.352	1.402	-
P16035	Metalloproteinase inhibitor 2 OS = Homo sapiens GN = TIMP2 PE = 1 SV = 2	1.440	1.459	1.426	1.438
P07996	Thrombospondin-1 OS = Homo sapiens GN = THBS1 PE = 1 SV = 2	1.433	1.454	1.337	1.303
P80188	Neutrophil gelatinase-associated lipocalin OS = Homo sapiens GN = LCN2 PE = 1 SV = 2	1.431	1.492	1.487	1.523
Q16270	Insulin-like growth factor-binding protein 7 OS = Homo sapiens GN = IGFBP7 PE = 1 SV = 1	1.425	1.434	1.381	1.376
Q14697	Neutral alpha-glucosidase AB OS = Homo sapiens GN = GANAB PE = 1 SV = 3	1.414	-	1.279	-
P04004	VTNC_HUMAN Vitronectin OS = Homo sapiens GN = VTN PE = 1 SV = 1	-	-	1.235	1.613
O00300	TR11B_HUMAN Tumor necrosis factor receptor superfamily member 11B OS = Homo sapiens GN = TNFRSF 11B PE = 1 SV = 3	-	1.342	-	1.409
P07858	Cathepsin B OS = Homo sapiens GN = CTSB PE = 1 SV = 3	1.214	1.420	1.214	1.313
P04179	Superoxide dismutase [Mn], mitochondrial OS = Homo sapiens GN = SOD2 PE = 1 SV = 2	0.789	0.707	0.789	0.708
Q5QNW6	Histone H2B type 2-F OS = Homo sapiens GN = HIST2H2BF PE = 1 SV = 3	0.739	0.668	0.588	0.706
P29401	Transketolase OS = Homo sapiens GN = TKT PE = 1 SV = 3	0.707	0.678	0.767	0.848
P02768	Serum albumin OS = Homo sapiens GN = ALB PE = 1 SV = 2	0.580	0.630	0.624	0.655
P62805	Histone H4 OS = Homo sapiens GN = HIST1H4A PE = 1 SV = 2	0.569	0.609	0.567	0.647
Q71DI3	Histone H3.2 OS = Homo sapiens GN = HIST2H3A PE = 1 SV = 3	0.543	0.528	0.558	0.594
P0C0S8	Histone H2A type 1 OS = Homo sapiens GN = HIST1H2AG PE = 1 SV = 2	0.443	0.513	0.461	0.513

**Figure 3:**
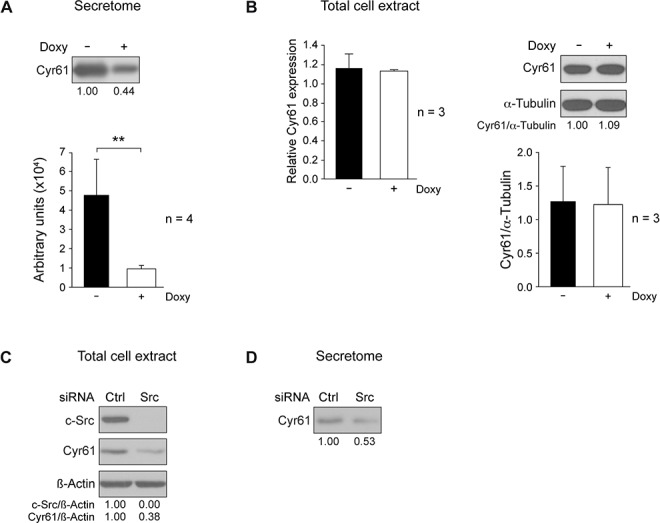
c-Src suppression reduces Cyr61 in MDA-MB-231-Tet-On-shRNA-c-Src and in SUM159PT cells **A.** Conditioned media from equal number of control or Doxy-treated MDA-MB-231-Tet-On-shRNA-c-Src were used to prepare secretome (S3 fraction) by centrifugation as in Figure 4A. After concentration by methanol/chloroform precipitation, pellet was used for detection of Cyr61 by immunoblotting. Quantitation of 4 independent experiments by ImageJ is shown. **B.** Analysis of Cyr61 mRNA and protein in total cell extracts from control and Doxy-treated (2 μg/ml) cultures grown for 72 h. Cyr61 (CCN1) mRNA expression was determined by qRT-PCR employing GAPDH as endogenous control (see Materials and Methods). Results are shown as mean ± SD of relative Cyr61 mRNA levels in three independent experiments in triplicate, considering arbitrarily the first sample of Doxy-untreated cells triplicate as 1. Intracellular Cyr61 was analyzed by immunoblotting, and α-tubulin was used for loading control. Quantitation of 3 independent experiments by ImageJ is shown. (**p < 0.01). **C.** c-Src was suppressed in SUM159PT cells by transient transfection of siRNA-hs-c-Src (see Materials and Methods), and levels of c-Src and Cyr61 were determined by immunoblotting in total cell extracts. **D.** Detection of Cyr61 levels in the secretome derived from equal number of SUM159PT cells after c-Src depletion.

The protein showing the highest reduction upon c-Src ablation, about 3.5 fold, was Cyr61 (Cysteine-rich protein 61, CCN1), an extracellular matrix-associated protein involved in migration and invasion of MDA-MB-231 cells [51]. Analyses of total secretome by immunoblotting confirmed reduced expression of Cyr61 in Doxy-treated cells (Figure 3A). To verify whether Doxy-treatment of wild-type MDA-MB-231 cells altered Cyr61 levels, we analyzed Cyr61 in the secretome, but no alterations were detected (data not shown). When chicken c-Src was constitutively expressed in c-Src suppressed MDA-MB-231 cells, the secreted Cyr61 levels were restored, confirming once again the specificity of the shRNA-c-Src (Supplementary Figure 1C).

Next, we evaluated whether c-Src suppression changed the intracellular levels of CCN1/Cyr61 mRNA and protein. Total RNA was isolated from exponentially growing cultures in presence or absence of Doxy (2 μg/ml for 72 h), and levels of mRNA and protein were determined by qRT-PCR and immunoblotting (see Materials and Methods). Surprisingly, suppression of c-Src did not seem to alter the intracellular content of mRNA and protein of CCN1/Cyr61 (Figure 3B). To support these observations, we analyzed the effects of c-Src suppression in another triple negative breast cancer cell line, SUM159PT. When c-Src was suppressed by means of a specific siRNA in SUM159PT (Figure 3C), the amount of Cyr61 was highly reduced in the secretome, as well as intracellularly (Figure 3C, D), in contrast to the results obtained in MDA-MB-231 cells.

Similarly to c-Src knockdown, conditional expression of SrcDN in MDA-MB-231 cells did not modify CCN1/Cyr61 mRNA and protein levels in cells (Supplementary Figure 3D), but it diminished the levels of Cyr61 in the secretome (Supplementary Figure 3E). Analogous results were obtained for SUM159PT after conditional expression of SrcDN (Supplementary Figure 3F).

**Figure 4:**
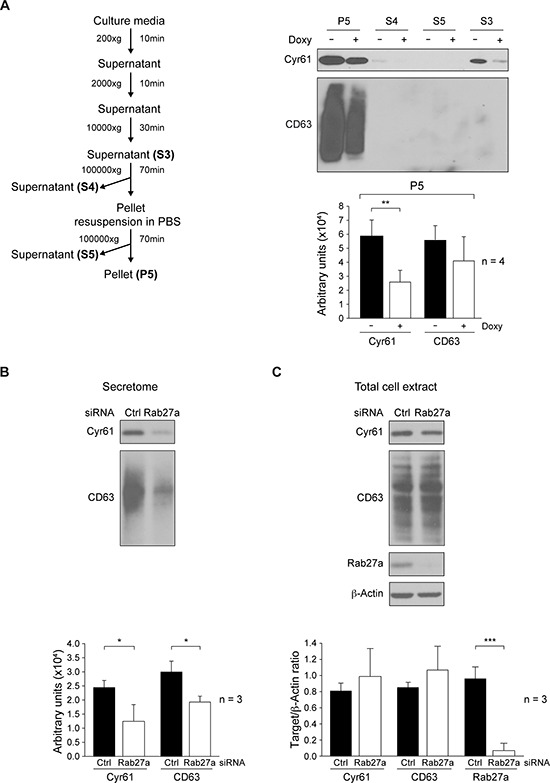
Analyses of cellular and secretome Cyr61distribution **A.** Fractionation of secretome by differential centrifugation from MDA-MB-231-Tet-On-shRNA-c-Src cultures grown in presence or absence of Doxy (2 μg/ml) for 72 h. After protein concentration by methanol/chloroform precipitation of fractions, expression of Cyr61 and CD63 was analyzed by immunoblotting. ImageJ densitometry quantification of four independent experiments (mean ± SD) is reported below and expressed in arbitrary units (**p < 0.01). **B., C.** MDA-MB-231 cells were transiently transfected with either scramble siRNA (Ctrl) or Rab27a-SiRNA (Rab27a) for 96 h. In secretome fraction S3, obtained from cultures with equal number of cells, and expression of Cyr61 and CD63 determined by immunoblotting **B.** Expression of Cyr61, CD63 and Rab27a was determined by immunoblotting in total cell extracts. Membranes were reblotted with anti-β-actin for loading control **C.** ImageJ densitometry quantification of three independent experiments (mean ± SD) is reported below and expressed as Target/β-actin ratio (for total cell extract) or in arbitrary units (for secretome) (*p < 0.05, ***p < 0.001).

To further characterize the mechanisms by which c-Src may influence Cyr61 secretion, we determined whether Cyr61 was a soluble protein or associated with the vesicular fraction of the secretome. Since suppression of c-Src in MDA-MB-231 cells did not alter cell proliferation or viability (Figure 1), conditioned media from equal numbers of untreated or Doxy-treated cells were fractionated using differential centrifugation [52]. Proteins from supernatants 3, 4 and 5 (S3, S4 and S5, see diagram of Figure 4A) were subsequently concentrated by methanol/chloroform precipitation, and together with pellet 5 (P5) analyzed by immunoblot to detect Cyr61. Results showed that Cyr61 was mainly found in P5. In addition, the exosomal marker tetraspanin CD63 [53] was exclusively detected in this fraction that contains exosomes [52]. Cyr61 was also weakly detected in soluble secretome of the S4 of control cells that were not treated with Doxy. Furthermore, statistical analyses of immunoblot-signals from four independent experiments showed that treatment with Doxy significantly reduced only expression of Cyr61 (Figure 4A).

To clarify the role of c-Src on exosomal protein secretion, the number of secreted vesicles was quantified in the exosomal fraction (P5) obtained from culture media of control and c-Src depleted MDA-MB-231 cells (see Supplementary Materials and Methods). Suppression of c-Src did not modify the number of total exocytic vesicles and of exosomes (size between 50 and 150 nm) (Supplementary Figure 4A and B, respectively), suggesting that c-Src does not modulate their secretion. Furthermore, the total protein concentration of fractions S3 and P5 were unaltered upon c-Src suppression (Supplementary Figure 4C). Moreover, siRNA suppression of Rab27a, which controls the exosomal secretion pathway [54], significantly reduced the expression of both Cyr61 and CD63 in the secretome (Figure 4B). However, it did not statistically alter their intracellular levels (Figure 4C). It is important to indicate that c-Src suppression did not modify cellular expression of Rab27a (Supplementary Figure 5). Furthermore, we observed that Cyr61 was mainly found in exosomes not only in MDA-MB-231, but also in SUM159PT, as transient expression of siRNA-c-Src in SUM159PT also showed a reduction of Cyr61 levels in exosomes (fraction P5), while CD63 levels remained unaltered (Supplementary Figure 6).

Additionally, we evaluated co-localization of Cyr61 with markers of secretory pathways, such as the cis-Golgi marker gp74 [55], and the late endosomal, lysosomal, and exosomal marker CD63 [53] in MDA-MB-231 cells. Analyses of confocal microscopy images showed a partial co-localization of Cyr61 with both gp74 and CD63 (Pearson's coefficient: 0.60 and 0.54, respectively) [56], as well as in Doxy-treated cells (Pearson's coefficient: 0.64 and 0.51, respectively) but no differences were observed between untreated and Doxy-treated cells (Supplementary Figure 7A, B). Together, these results suggest that c-Src controls levels of exosomal Cyr61.

### Silencing Cyr61 reduced invasion and transendothelial migration

**Figure 5:**
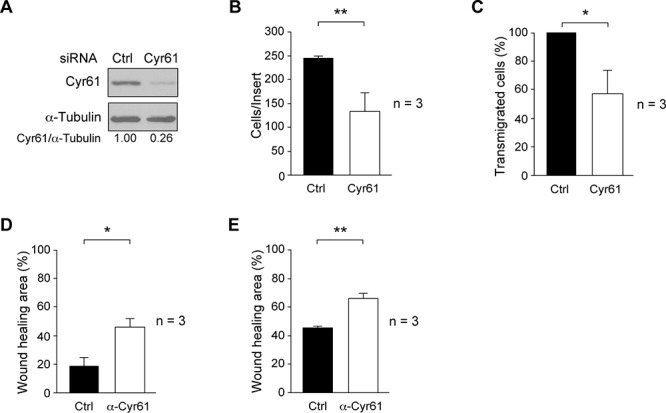
Effect of Cyr61 suppression or antibody neutralization on breast cancer cell motility properties **A.** MDA-MB-231 were transiently transfected with siRNA-Cyr61 for 72 h and, Cyr61 expression determined from total cell extracts by immunoblotting. Membranes were reblotted with anti-α-tubulin for loading control. Results are representative of three independent experiments in triplicate. **B.** For cell invasion through Matrigel-coated inserts, 48 h after siRNA-Cyr61 or siRNA-Control transfected cells were loaded onto Matrigel. After 22 h, invaded cells were fixed, stained with DAPI and counted by fluorescence microcopy. The number of invaded cells per insert is shown and represents average ± SD of three experiments performed in triplicate (**p < 0.01). **C.** For transendothelial migration, cells were seeded onto the HUVEC monolayer, and 48 h after siRNA-Cyr61 transfection. Transmigrated cells were detached after 22 h and counted in a hemocytometer. The number of siRNA-Cyr61 transmigrated cells was expressed as percentage of siRNA-Control transmigrated cells (100%). **D.** Cultures of MDA-MB-231-Tet-On-shRNA-c-Src in absence of Doxy were treated with 4 μg/ml of anti-Cyr61 (Cyr61) or with the corresponding amount of normal rabbit serum (Ctrl) for 48 h. **E.** Cultures of SUM159PT were treated with 1 μg/ml of anti-Cyr61 or with the corresponding amount of normal rabbit serum (Ctrl) for the last 20 h. Analyses of wound-healing were made at 0 and 20 h as described in Materials and Methods. Results are expressed as mean percentage of wound healing area ± SD at 20 h respect to 0 h from three independent experiments performed in triplicate (*p < 0.05, **p < 0.01).

The above data indicate that c-Src suppression reduced invasiveness and transendothelial migration, which are properties of metastatic cells, and also diminished the levels of Cyr61 in the secretome. To elucidate whether Cyr61 mediated c-Src biological effects in MDA-MB-231 cells, we investigated the cellular invasiveness and transendothelial migration of cells following the siRNA-mediated depletion of Cyr61. Exponentially growing MDA-MB-231 cultures were transiently transfected with scramble-siRNA (Ctrl) and Cyr61-siRNA as described in Materials and Methods. Compared to control siRNA, specific Cyr61 siRNA significantly reduced Cyr61 expression (Figure 5A), as well as invasion and transendothelial migration (Figure 5B, C).

### Neutralization of Cyr61 reduced cell migration

Furthermore, to determine if secreted Cyr61 had a role in cell migration, cultures of MDA-MB-231-Tet-On-shRNA-c-Src or SUM159PT were incubated with either Cyr61 antibody (α-Cyr61) or with the corresponding amount of normal rabbit serum (Ctrl) in wound-healing assays. Antibody neutralization of Cyr61 significantly reduced cell migration as compared with the control (normal rabbit serum) both in MDA-MB-231-Tet-On-shRNA-c-Src and SUM159PT cell lines (Figure 5D, E, respectively).

These results support the role of Cyr61 as a mediator of c-Src signaling in the regulation of these cellular events, which are associated with metastatic properties of triple negative breast cancer cells.

### Expression of Cyr61 in breast cancer

**Figure 6:**
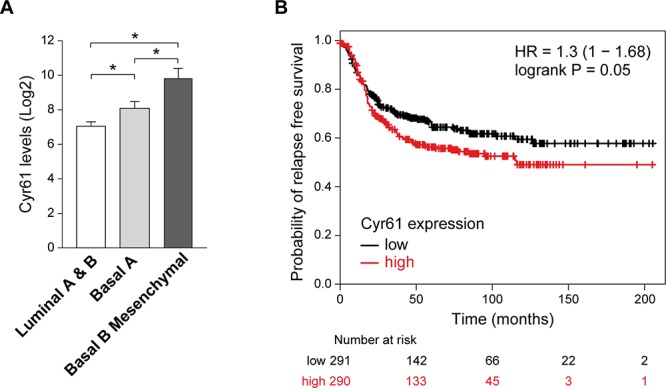
Expression of Cyr61 in breast cancer **A.** Different expression levels of *Cyr61* in human cell lines of breast cancer of Luminal A (ER +), Luminal B (ER-), Basal A, and Basal B (i.e. with epithelial to mesenchymal transition). Notice that the last group shows the higher levels of *Cyr61* expression. Below, *Cyr61* is significantly expressed at higher levels in cell lines that had undergone epithelial to mesenchymal transition. Figure generated from the available database [59]. Comparison among groups was done by ANOVA followed by Tukey post-hoc test. **B.** Levels of *Cyr61* expression define tumor prognosis of patients with breast cancer of basal origin. Figure generated with the Kaplan-Meier plotter available at the web page: http://kmplot.com/analysis/index.php?p=service from the database [62].

Cyr61 has been involved in breast cancer [51, 57], its expression correlates with lack of estrogen receptor [58]. To determine whether there is a relationship between Cyr61 expression and the different breast cancer subtypes (Luminal A and B, Basal A and Basal B), we analyzed its levels in 51 human breast cancer cell lines according to published data [59]. The results showed that the lowest levels of Cyr61 were found in Luminal A and B subtypes (Figure 6A). Furthermore, it was differentially expressed in ER^−^ breast cancer subtypes, since Cyr61 mRNA levels was higher in Basal B than in Basal A subtype. Basal B cell lines, like MDA-MB-231 or SUM159PT have a mesenchymal-like phenotype and features of cancer stem cells (CD44^+^ CD24^−/low^, ALDH1^+^) [60], indicating that Cyr61 is associated with a more aggressive phenotype. In fact, analyses of 581 human samples of Basal-like tumors [St Gallen International Expert Consensus on the Primary Therapy of Early Breast Cancer 2011 [61]] by Kaplan-Meier plotter based on RFS (Relapse Free Survival) [62], showed that high expression of Cyr61 was significantly associated to lower probability of RFS (Figure 6B).

## DISCUSSION

SFKs participate in the development and progression of several human cancers, including breast cancer. Furthermore, SFKs are involved in MDA-MB-231 metastasis to bone, lung [47, 48] and brain [49]. To investigate the role of c-Src in the first steps that lead to metastasis, we generated three inducible cell lines, MDA-MB-231-Tet-On-shRNA-c-Src, MDA-MB-231-Tet-On-SrcDN, and SUM159PT-Tet-On-SrcDN. In addition, c-Src was transiently suppressed in SUM159PT cells. We found that c-Src modulates migration, invasion and transendothelial migration processes by regulating the phosphorylation of focal adhesion proteins along with the levels of secreted proteins such us IGFBP4, CTGF, and Cyr61, a new exosomal protein involved in tumor progression.

An essential property of metastatic cells is their capacity to grow in the absence of a substrate. Our findings show that c-Src suppression significantly reduced the capacity of MDA-MB-231 cells to grow as colonies in soft agar. Previous results support the role of c-Src in anchorage-independent growth. Silencing of c-Src, but not Yes or Fyn, reduced the ability to form colonies in MDA-MB-231, MDA-MB-436 and SKBR3 cells [63]. Furthermore, SFKs catalytic activity inhibition or stable transfection of catalytically inactive c-Src into MDA-MB-468 and MCF7 reduced colony formation ability [33]. Therefore, c-Src is required for breast cancer cell growth in anchorage-independent conditions.

SFKs participate in focal adhesion turnover that control cell motility and, in turn, cell migration and invasion. Fak/Src complex phosphorylates several proteins including p130CAS, paxillin or caveolin 1 promoting focal adhesion turnover [64, 65]. c-Src suppression decreased cell migration and invasion, as well as diminished phosphorylation of Y-p130CAS, Y118-paxillin, and Y14-caveolin 1. Additionally, SrcDN expression also reduced cell migration and invasion. SFKs catalytic activity inhibition provoked comparable effects on MDA-MB-231 cells [31]. Analogous results were obtained after SrcDN constitutive expression in MDA-MB-231 cells [41]. In MCF7 cells constitutive suppression of c-Src or SrcDN conditional expression reduced cell migration [36]. Matrix metalloproteases degrade extracellular matrix proteins and, therefore, are necessary for cell invasion [66]. We also observed that c-Src depletion or SrcDN expression in MDA-MB-231 reduced the levels of the matrix metalloproteases MMP2, MMP7, and MMP9 in the secretome. Previous observations show that SFKs catalytic activity inhibition by PP2 in MDA-MB-231 cells decreased MMP9 production, as well as invadopodia formation [67]. Besides, PH006, another SFKs catalytic activity inhibitor, reduced MMP2 and MMP9 secretion and invasion of MDA-MB-231 cells [68]. These findings suggest that c-Src is essential for cell migration and invasion.

To further characterize the contribution of c-Src in both events we analyzed the secretome of c-Src-depleted MDA-MB-231 cells. Twenty proteins were differentially expressed, 13 were down-regulated and 7 up-regulated. Among the down-regulated proteins, we found Cysteine-rich protein 61 (Cyr61), Insulin-like growth factor-binding protein 4 (IGBP4), and Connective tissue growth factor (CTGF). However, MMPs were undetected in the proteomic analyses because the immunoblotting was more sensitive under the experimental design employed (see Materials and Methods). The most differentially down-regulated protein was Cyr61, a CCN family member that participates in cell migration, differentiation, proliferation, adhesion, and angiogenesis [69]. The reduction in secreted Cyr61 did not correspond to the unaltered intracellular mRNA and protein levels observed in c-Src-depleted MDA-MB-231 cells. Furthermore, this is not an off-target effect of the shRNA-c-Src, since the constitutive expression of chicken c-Src restored Cyr61 levels in the secretome.

It is known that Cyr61 is highly expressed in invasive breast cancer cell lines, and its expression correlates with the absence of estrogen receptor [58]. We observed the lowest levels in Luminal A and B subtypes and the highest in Basal cells. Moreover, the most aggressive basal-like subtype, Basal B, has significantly higher levels of Cyr61 than Basal A. Cyr61 was expressed in 36% of primary breast tumors [70] and in about 30% of invasive breast carcinomas. High Cyr61 levels are related to advance stage and tumor size [71, 72]. We found that higher Cyr61 expression significantly reduced probability of relapse free survival, by the analyses of 581 human samples of basal-like tumors. Therefore, Cyr61 could be a marker of poor prognosis in basal breast carcinoma.

In MDA-MB-231 cells constitutive expression of Sonic Hh (SHH) increased secreted Cyr61. Besides, silencing of Cyr61 reduced invasion of SHH-expressing MDA-MB-231 cells, as well as diminished the ability to induce endothelial tube. Moreover, Cyr61 knockdown reduced tumor growth [73]. The ectopic expression of Cyr61 in MCF7 promoted estrogen-independent growth, and its overexpression increases tumors growth, as well as, density of blood vessel in tumors [70, 74]. We observed diminished invasive and transendothelial migration abilities after c-Src suppression, as well as, after transient silencing of Cyr61 in MDA-MB-231 cells. Moreover, neutralization of secreted Cyr61 reduced cell migration in MDA-MB-231 and SUM159PT cells. Similarly, Cyr61 neutralization reduced MDA-MB-231 migration and invasion *in vivo* and *in vitro* [51]. Together, these data support the role of Cyr61 as a mediator, at least in part, for the role of c-Src in invasion and extravasation.

Cyr61 is associated with the extracellular matrix and we found a small portion in soluble secretome. However, Cyr61 was mainly present in the exosomal fraction. Knockdown of Rab27a, a small GTPase involved in exosomal secretion [54], resulted in reduced levels of CD63 and Cyr61 in the secretome. We observed a partial co-localization of Cyr61 with markers of the secretory pathway, including the cis-Golgi marker gp74 [55], as well as, CD63, a marker of late endosomes, lysosomes and exosomes [75, 76]. However, we could not discriminate the effects of c-Src on this part of the secretory pathway. Furthermore, the reduced levels of Cyr61, MMP2, MMP7 and MMP9 in the secretome upon c-Src suppression in MDA-MB-231 cells is not a general effect of this proto-oncogene on protein secretion, as the total number of exocytic vesicles and exosomes was not modified, nor was the protein concentration of fraction S3 and P5. In view of the results, we could hypothesize that the absence of c-Src might favor Cyr61 proteolysis in the secretome by protease activation. c-Src suppression reduced intracellular Cyr61 in SUM159PT, concomitantly with an increase in the cysteine protease cathepsin F, not observed in MDA-MB-231 (data not shown). Moreover, Src family kinase activity inhibition by Dasatinib or PP2 in MDA-MB-231 also diminished intracellular Cyr61 levels (data not shown), while cathepsin F mRNA was increased [31]. Indeed, we observed that the levels of cystatin C, an inhibitor of cysteine proteases, were reduced in the secretome of c-Src-depleted MDA-MB-231 cells. Then, further studies are required to determine the molecular mechanisms by which c-Src controls secreted Cyr61.

Exosomes transfer information and act locally on cancer cells and stroma, or distantly to prepare niche for cancer cell implantation. Melanoma-derived exosomes promote metastatic niche formation through modification of bone marrow-derived cells. Exosomes from a metastatic melanoma cell line injected in mice localized to common sites of melanoma metastasis such as, lung, bone marrow, liver, and spleen [77]. Cyr61 is involved in bone remodeling, acting on osteoblast differentiation [78, 79] and its silencing in osteosarcoma tumors reduced vascularization and metastases to lung [80]. Then, we cannot discard its contribution to lung and bone metastasis of breast cancer cells. Furthermore, an up-regulation of Cyr61 and CTGF was observed in bone-derived MDA-MB-231 cells compared to parental MDA-MB-231 cells [81]. CTGF, another CCN member, participates in osteolytic metastasis of highly aggressive bone-derived MDA-MB-231 population [82]. Moreover, CTGF-integrin αvβ3-Erk1/2 pathway regulates S100A4 gene that contributes to metastatic ability of MDA-MB-231 cells in a lung metastatic mouse model [83]. Therefore, c-Src might alter metastatic potential of triple negative breast cancer cells by modulating secreted proteins including Cyr61 and CTGF.

In conclusion c-Src modulation may be essential to breast cancer metastasis, since regulates MDA-MB-231 cell survival in absence of substrate. Besides, c-Src modulates invasion, migration, and transendothelial migration, essential processes in metastatic cascade, by controlling secreted proteins, particularly the new exosomal protein, Cyr61.

## MATERIALS AND METHODS

### Reagents

Anti-c-Src MAb-327 [84], provided by J.S. Brugge, Harvard University. Anti-Fak, anti-Cyr61, and anti-cyclin D1 were from Santa Cruz Biotechnology. Anti-CD63 (Inmuno-Step; Calbiochem). Antibodies to MMP2, MMP9, and MAb 4G10 were from Merk-Millipore. Anti-MMP7 was from Abgent. Anti-pY397-Fak, secondary horseradish peroxidase-conjugated antibodies, siRNA-hs-Cyr61 (s7244, Silencer^®^ selected and validated siRNA), siRNA-hs-c-Src (s13414, Silencer^®^ selected and validated siRNA), and scramble siRNA (Stealth RNAi Negative Control Duplex #12935–300) were from Life Technologies. Anti-pY925-Fak was from Cell Signaling Technologies. Anti-paxillin, anti-pY118-paxillin, anti-p130CAS, anti-caveolin-1, anti-pY14-caveolin-1, anti-p27^Kip1^, and Matrigel^TM^ were from BD-Biosciences. Anti-α-tubulin, anti-ß-actin, doxycycline (Doxy), Trypan blue, Thiazolyl Blue Tetrazolium Bromide (MTT reagent), and esiRNA human Rab27a were from Sigma-Aldrich. Anti-Rab27a polyclonal antibody (Peter van der Sluijs, University Utrecht, [85]). Anti-Tet-repressor (Mobitec). Blasticidin and zeocin were from InvivoGen. Tet-Free-FCS (PAA Laboratories GmbH). Acrylamide/Bis-acrylamide (29:1), SDS and ammonium persulfate were from Bio-Rad Laboratories. ECL was from GE Healthcare Biosciences. BCA protein assay and DharmaFECT 4 were from Thermo Scientific.

### Generation of MDA-MB-231 cell line with conditional expression (Tet-On) of shRNA-c-Src

Genotyped MDA-MB-231 (ATCC, HTB-26) cultured in DMEM containing 5% Tet-free FCS, 2mM glutamine, 100IU/ml penicillin, and 100 μg/ml streptomycin were transfected with pcDNA6/TR (Life Technologies) and selected with 6 μg/ml of blasticidin. Clones were grown and tested for maximal expression of TetR by immunoblotting. Clone TR8 was transfected with pENTR™/H1/TO containing the highly specific shRNA-c-Src: 5′-GCCTCAACGTGAAGCACTACA-3′ (100% identity and E-value 0.006 for mRNA transcripts variants 1 and 2, NM-198291 and NM-005417.4, of human c-Src by BLAST analysis) (Life-Technologies). After 48 h, cells were selected with blasticidin and 300 μg/ml of zeocin. Clones were grown −/+2 μg/ml Doxy for 72 h, and tested for c-Src expression by immunoblotting. Clones with high reduction of c-Src expression were pooled. Then, total RNA and protein were isolated from cells grown −/+ Doxy for different times and tested for c-Src mRNA by qRT-PCR and protein expression by immunoblotting.

### Transient transfection of siRNA-c-Src in SUM159PT

The triple negative human breast cancer cells SUM159PT [86], provided by G. Dontu [87], were propagated in Ham's F12, 5% FCS, 5 μg/ml insulin, 1 μg/ml hydrocortisone, 2mM glutamine, 100IU/ml penicillin, and 100 μg/ml streptomycin. For transient transfection, SUM159PT cells (1.5 × 10^5^cells/well, 6-well plate) were seeded in complete medium without antibiotics. Next day, cells were transfected with 20nM of siRNA-hs-c-Src or 20nM scramble siRNA for control, using Dharmafect 4. After 8 h medium was changed, and cells were cultured for 48 h, then the media was replaced for serum-free media, and cultures maintained for another 24 h. Secretome and total cell extracts were used for immunoblotting analyses.

### Metabolic activity and cell viability studies

For MTT assay, cells were incubated in the presence or absence of Doxy (2 μg/ml) for 72 h. Then, 10 μl of MTT reagent were added to each well (final concentration 0.5mg/ml) and plates were incubated at 37°C for 4 h. After solubilization of formazan crystals (2–3 h at 37°C with 10%SDS/10mM HCl), absorbance was measured at 570nm in a VersaMax Elisa Microplate Reader (Molecular Devices). Cell viability was determined counting cells after Trypan blue labeling. Control and Doxy-treated cells (2 μg/ml, 72 h) were detached, mixed with a 0.4% Trypan blue/PBS solution (1:1), loaded on a hemocytometer and counted.

### Anchorage-independent growth

Cells were resuspended in warmed solution of 0.3% agarose in complete medium −/+2 μg/ml Doxy and seeded at 10^5^cells/60mm dishes with a bottom layer of 0.5% agarose. Cells were re-fed every 3 days with complete medium (300μl/dish, −/+Doxy). Plates were stained with 0.5 ml of 0.005% crystal violet/water for 1 h. Colonies with diameter ≥ 0.1mm were counted after 20 days. Three independent experiments were performed in triplicate.

### Immunoblotting

Cell lysed from three subconfluent plates were analyzed by immunoblotting as previously described [36].

### RNA preparation, qRT-PCR of mRNA

Cell cultures of MDA-MB-231-Tet-On-shRNA-c-Src were incubated −/+ Doxy (2 μg/ml) for 72 h. RNA was isolated from three independent experiments performed in triplicate using RNeasy kit (Qiagen). After testing for RNA integrity, triplicate RNAs from each treatment/experiment were pooled. c-Src mRNA expression was determined by using TaqMan probe for human c-Src (Hs01082246_m1), TBP (TATA-box Binding Protein) was used as endogenous control (Life Technologies). For CCN1 (Cyr61) qRT-PCR was performed using SYBR Green Master Mix (Life Technologies). Specific primers for Cyr61 and GAPDH, used as endogenous control, were previously described [88]. Gene expression was calculated as difference in cycle threshold (ΔCt) between target gene and GAPDH and TBP respectively; ΔΔCt was the difference between ΔCt values of test sample and that of control. Relative expression of target genes was calculated as 2^−ΔΔCt^.

### Cell migration

MDA-MB-231-Tet-On-shRNA-c-Src cells were seeded in complete medium (3.5 × 10^5^cells/well in 6-well plate) and grown to confluence (48 h, −/+2 μg/ml Doxy). SUM159PT cells were seeded in complete medium (2.25 × 10^5^cells/well in 6-well plate) and grown to confluence 24 h. The monolayer was scratched with a 200μl micropipette tip and washed with fresh medium to remove floating cells. Complete medium was added −/+ Doxy to MDA-MB-231-Tet-On-shRNA-c-Src. To test for the biological role of secreted Cyr61 in migration, cultures of MDA-MB-231-Tet-On-shRNA-c-Src in absence of Doxy were treated with 4 μg/ml of anti-Cyr61 (Cyr61) or with the corresponding amount of normal rabbit serum (Ctrl) for 48 h. For SUM159PT complete medium −/+1 μg/ml neutralizing antibody Cyr61 was added to cultures. Photomicrographs were taken at 0 and 20 h with a Microscope Cell Observer Z1 system (Carl Zeiss AG) equipped with controlled environment chamber and Camera Cascade 1k. Migration was quantified using wound-healing tool of ImageJ.

### Transendothelial migration

Transendothelial migration of MDA-MB-231-Tet-On-shRNA-c-Src was performed growing HUVEC cells over gelatin-coated cell culture inserts for 24-well plate (8 μm-pore PET membranes, Falcon). After 48 h −/+Doxy, 5 × 10^4^cells were seeded over HUVEC monolayer. The lower chamber was filled with 600μl of 20% FBS-supplemented medium. In parallel, a control was performed in the same conditions by seeding HUVEC cells alone, in order to test their spontaneous migration. After 22 h, transmigrated cells were detached and counted in hemocytometer. The number of Doxy-treated transmigrated cells was expressed as percentage respect to control transmigrated cells (100%).

### Invasion assay

MDA-MB-231-Tet-On-shRNA-c-Src cells were seeded on the upper chamber (5 × 10^4^/well/200μl) in serum-free medium −/+2 μg/ml Doxy. The lower chamber was filled with 600μl of 20% FBS-supplemented medium −/+ Doxy; 22 h later, after removing cells on top of inserts, those on lower surface fixed and stained as previously described [31].

### Secretome fractionation

Conditioned media from cell cultures were used to prepare soluble and exosomal fractions of secretome by differential centrifugation, as described [52]. The soluble secretome, supernatant of 100, 000xg, 70min, was concentrated by methanol/chloroform precipitation. Microvesicle pellet was resuspended in RIPA buffer for immunoblotting analyses.

### Transient transfection of siRNA for Cyr61and Rab27a

MDA-MB-231 cells (10^5^cells/well, 6-well plate) were seeded in complete medium without antibiotics. Next day, cells were transfected using Dharmafect 4 with 20nM of siRNA-Cyr61 or 50nM of esiRNA-Rab27a. For control, 20/50nM scramble siRNA (Life Technologies) were used. After 8 h medium was changed. For siRNA-Cyr61 transfection, cells were detached 40 h later and used for invasion, transendothelial migration or immunoblotting (72 h) assays. When esiRNA-Rab27a was employed, media culture was changed after 72 h and cells were then maintained 24 h in serum-free media. Secretome and total cell extracts were used for immunoblotting analyses.

### Secretome protein digestion, iTRAQ-4-plex^®^ labeling and RP-LC-MALDI TOF/TOF MS analysis

Cultures of MDA-MB-231-shRNA-c-Src were grown for 48 h in complete media −/+ Doxy (2 μg/ml). Cultures were then washed with phenol red and serum free DMEM and incubated for additional 24 h in this medium −/+ Doxy (2 μg/ml). Total secretome, fraction S3 (diagram Figure 4A) was collected. For digestion, 20 μg of protein from each secretoma condition (−/+ Doxy) was precipitated with methanol/chloroform. Proteins from each condition (20 μg) were reduced with TCEP [Tris(2-carboxyethyl)phosphine], alkylated with MMTS (methyl methanethiosulfonate), digested with trypsin and labeled with iTRAQ reagent. Labeling tags 116 and 117 were used for controls and 114 and 115 for Doxy-treated replicates. Each digested, labeled and pooled samples were desalted using Sep-Pak C18 Cartridges (Waters), vacuum concentrated and reconstituted with 0.1% heptafluorobutyric acid (HFBA). An aliquot was injected with a TEMPO nanoMDLC HPLC (AB SCIEX) and eluted onto an Onyx monolith C18 column (150 mm × 0.1 mm) (Phenomenex). α-cyano-4-hidro-cinnamic acid MALDI matrix diluted in 70% acetonitrile and 0.1%TFA aqueous solution, were mixed post-column with eluted peptides. Spots were deposited on MALDI-plate (Opti-TOF™ LC MALDI Insert, AB SCIEX). A MALDI TOF/TOF 4800 (AB SCIEX) mass spectrometer was used for acquisition and processing of data. MS data from spots was acquired in positive reflector ion mode in mass range of 800–3500m/z by accumulation of 1200 laser shots. MS/MS spectra were generated by 2kV collisions with air. Maximal 2000 laser shots were accumulated for MS/MS spectra. Protein identification and quantitation on each of the two data sets were done by using MASCOT-v2.3.01 (Matrix Science) and Phenyx-v2.6 (GeneBio) search engines. Searches were performed against UniProtKB/Swiss-Prot human database to estimate false positive rate (FDR) below 0.5%, which boosted reliability of data. Differential expression of proteins between controls and Doxy-treated samples was determined by calculating weighted average ratios of peptides for each identified protein. Only proteins having at least two quantitated peptides detected by both Mascot and Phenyx in control and + Doxy conditions were considered in quantitation.

### Statistical analyses

Mean values, standard deviation and statistical significance between data from two different experimental conditions were determined by two-tail Student *t*-test. For co-localization analyses by confocal microscopy, Pearson's coefficient (Bolte and Cordelieres, 2006) [56] was employed. ANOVA followed by Tukey post-hoc test were used for comparative analyses of Cyr61 expression among subtypes of breast cancer cell lines.

## SUPPLEMENTARY FIGURES AND TABLES



## Abbreviations

Cyr61: Cysteine-rich protein 61, CCN1
DAPI: 4′, 6-diamidino-2-phenylindole
Doxy: Doxycycline
EGF: Epidermal Growth Factor
bFGF: basic Fibroblast Growth Factor
MTT: 3-(4, 5-dimethylthiazol-2-yl)-2, 5-diphenyltetrazolium bromide
PI: propidium iodide
RFS: relapse free survival
SFKs: Src Family Kinases
