# Supplementary material for: Cyr61 as mediator of Src signaling in triple negative breast cancer cells
Source: Oncotarget. 2015 Apr 20;6(15):13520–38. doi: 10.18632/oncotarget.3760 (PMC4537031; doi:10.18632/oncotarget.3760)
Supplement: Supplementary file 1 [file oncotarget-06-13520-s001.pdf]

SUPPLEMENTARY MATERIALS AND METHODS

GENERATION OF MDA-MB-231-TET-ON-SHRNA-C-SRC WITH CONSTITUTIVE EXPRESSION OF WILD-TYPE CHICKEN C-SRC

MDA-MB-231-Tet-On-shRNA-c-Src cells were transfected with pBABE-Puromycin containing chicken c-Src. After 48 h, cells were selected by growing in the presence of puromycin (0.5 µg/ml), in addition of blasticidin (6 µg/ml) and zeocin (300 µg/ml) to maintain the selection for the Tet-On shRNA-c-Src expression. A week later, clones were collected and pooled. This new cell line MDA-MB-231-Tet-On-shRNA-c-Src + chicken c-Src was tested for expression of chicken c-Src by immunoblotting with the MAb EC10, which specifically recognizes chicken c-Src.

RANDOM MIGRATION

Cells (5×10^4/well in 6-well plate) were seeding in complete medium. After 48 h of Doxy (2 µg/ml) treatment, medium was changed by 2% serum-medium. Photographs were taken every 15min for 22 h at 10x with Microscope Cell Observer Z1 system. Analysis was performed by manual tracking, chemotaxis, and migration tools of ImageJ ($n = 63$) to obtain the accumulated distance (length path) and mean velocity (length path/time). Assays were repeated 3 times.

IMMUNOFLUORESCENCE BY LASER-SCANNING CONFOCAL MICROSCOPY

Cells seeded on sterile coverslips were 72 h-treated with 2 µg/ml Doxy, fixed with 2% paraformaldehyde/PBS (15 min, room temperature), permeabilized with 0.5% Triton X100/PBS (10 min, room temperature) and blocked 1 h with 10% normal goat serum/PBS. Coverslips were incubated overnight at 4°C with primary antibodies diluted 1:100 in 5% normal goat serum/PBS: rabbit polyclonal anti-Cyr61 in combination with mouse monoclonal anti-cis-Golgi-gp74 25H8 (Ignacio Sandoval, CBMSO, Madrid) or anti-CD63. Coverslips were washed with PBS and then incubated for 1 h at 37°C with secondary antibodies diluted 1:400 in 5% normal goat serum/PBS: goat-anti-mouse IgG Alexa-Fluor 488 or goat-anti-rabbit IgG Alexa-Fluor 546 (Life Technologies). After washing with PBS, cells were counterstained with DAPI (D1306, Life Technologies) in PBS (10 µg/ml) for 5min at room temperature, washed in PBS and coverslips mounted on slides with ProLong reagent (Life Technologies). Samples were analyzed by confocal microscopy. Images were acquired using an inverted Zeiss LSM 710 laser-scanning microscope with a Plan Apochromat 60x/1.40 objective. Sequential scanning mode was used to avoid crosstalk between channels. Z-optical stacks with 0.6 µm intervals through the cell Z-axis were recorded. Images were processed with ZEN 2009 software (Carl Zeiss AG) and Adobe Photoshop CS5 (Adobe Systems In.). To determine co-localization of antigens, confocal images were analyzed by JACoP of ImageJ obtaining Pearson's coefficients [1].

GENERATION OF MDA-MB-231 AND SUM159PT CELL LINES WITH CONDITIONAL EXPRESSION (TET-ON) OF SRCDN

MDA-MB-231 cells and SUM159PT were transfected with pcDNA6/TR (Life Technologies) and clones were isolated with 6 µg/ml of blasticidin. Clones were grown and tested for maximal expression of TetR by immunoblotting. Clone TR8 was selected and transfected with pcDNA4/TO containing the chicken c-Src mutant SrcDN (c-Src K295A/Y527F). After 48 h, cells were selected for growing in the presence of blasticidin (6 µg/ml) and of zeocin (300 µg/ml). Clones were grown in absence or presence of doxycycline (Doxy, 2 µg/ml) for 72 h and tested for the expression of c-Src by immunoblotting with EC10 mAb (Merck-Millipore) that specifically recognize chicken c-Src. Clones with a significant SrcDN expression were selected and pooled. MDA-MB-231-Tet-On-SrcDN were maintained in DMEM, SUM159PT were cultured in Ham's F12, 5 µg/ml insulin, 1 µg/ml hydrocortisone, both cell culture media were supplemented with 5% Tet-Free-FCS, 2 mM glutamine, 100IU/ml penicillin, 100 µg/ml streptomycin, 3 µg/ml blasticidin and 100 µg/ml zeocin.

SUPPLEMENTARY REFERENCES

1. Bolte S, Cordelieres FP. A guided tour into subcellular colocalization analysis in light microscopy. Journal of microscopy. 2006; 224:213–232.

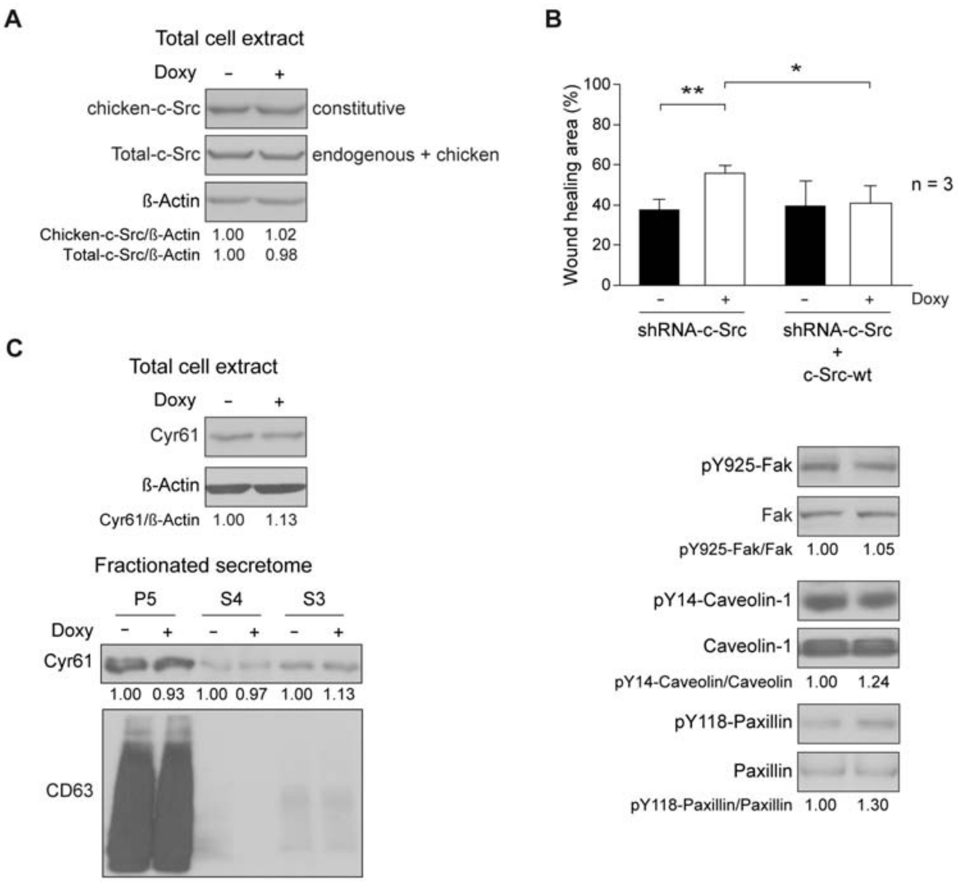

Supplementary Figure S1: Specificity of the shRNA for human c-Src in MDA-MB-231. To test for specificity of the shRNA-c-Src, MDA-MB-231-Tet-On-shRNA-c-Src cells were transfected with chicken c-Src cloned into pBABE-Puromycin. Puromycin-resistant clones were pooled and **A.** total cell extracts were tested by immunoblotting for expression of chicken c-Src with the specific MAb EC10 and to total c-Src with MAb 327 in untreated and Doxy-treated cultures (2 µg/ml for 72 h). **B.** Wound-healing migration assay of MDA-MB-231-Tet-On-shRNA-c-Src and MDA-MB-231-Tet-On-shRNA-c-Src+c-Src-wt in untreated or Doxy-treated cultures, and immunoblotting analyses of the activation/phosphorylation of Fak (Y925), caveolin-1 (Y14), and paxillin (Y118). Membranes were reblotted with the corresponding antibodies for Fak, caveolin, and paxillin for loading controls. **C.** Immunoblotting detection of Cyr61 levels in total cell extracts and fractions from the secretome.

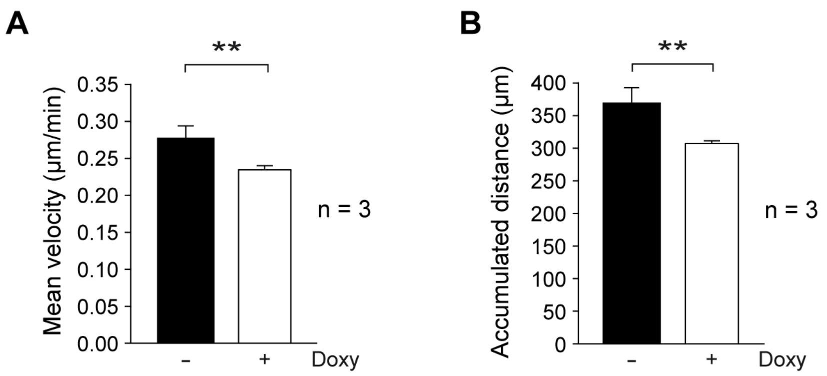

Supplementary Figure S2: Random-migration assay in sub-confluent MDA-MB-231-Tet-On-shRNA-c-Src cultures (control and 48 h-Doxy treated). The mean velocity **A.** and the accumulated distance **B.** from three independent experiments were calculated by photographs analysis by ImageJ tools (**p < 0.01).

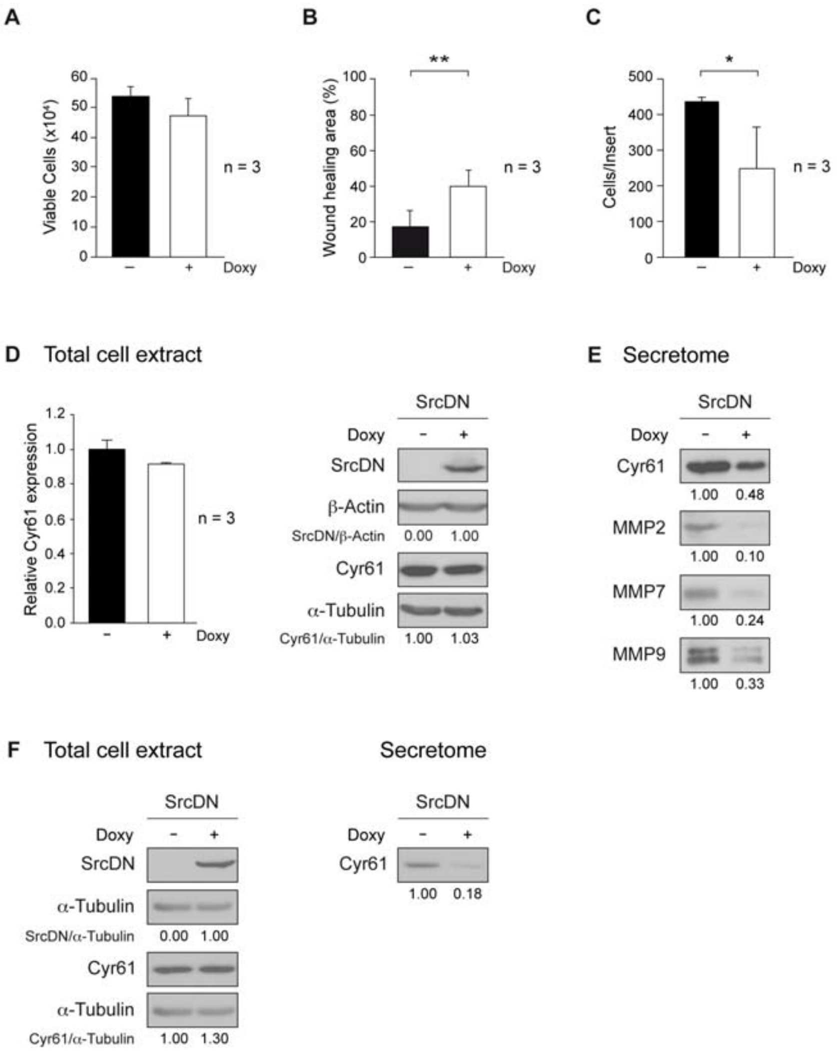

Supplementary Figure S3: Effect of SrcDN conditional expression on MDA-MB-231 and SUM159PT cells. MDA-MB-231-Tet-On-SrcDN cells (A–E) were grown with or without Doxy (2 μg/ml) for 72 h. **A.** Cell viability was evaluated counting cells after Trypan blue labeling. Results are shown as mean ± SD from three independent experiments in triplicate. **B.** Cell migration was determined by wound-healing assay through scratching confluent cultures; photomicrographs were taken every 30min for 20 h with a Microscope Cell Observer Z1 system, and quantified using wound-healing tool of ImageJ. Results are expressed as mean percentage of wound healing area ± SD at 20 h respect to 0 h from three independent experiments (**$p < 0.01$). **C.** For cell invasion assays through Matrigel-coated inserts, cultures were grown for 48 h -/+ 2 μg/ml Doxy and then seeded onto Matrigel (-/+ 2 μg/ml Doxy); 22 h later, cells on the top of inserts were removed and invaded cells were fixed, stained with DAPI and counted by fluorescence microscopy. The number of invaded cells per insert is shown and represents average ± SD of four experiments in triplicate (*$p < 0.05$). **D.** Total RNA and protein were isolated from cells grown with or without Doxy (2 μg/ml) for 72 h. Cyr61 (CCN1) mRNA expression was determined by qRT-PCR employing GAPDH as endogenous control (see Materials and methods). Results are shown as mean ± SD of relative Cyr61 mRNA levels in three independent experiments in triplicate, considering arbitrarily the first sample of Doxy-untreated cells as 1 (see Materials and Methods). Results are shown as mean ± SD of relative c-Src mRNA levels in three independent experiments in triplicate. Cell extracts from three subconfluent p60 plates grown with or without Doxy (2 μg/ml) for 72 h were pooled and used to detect SrcDN and Cyr61 by immunoblotting with MAb-EC10 or anti-Cyr61, respectively; membranes were then reblotted with anti-β-actin or anti-α-tubulin respectively for loading control. Results are representative of three independent experiments in triplicate. **E.** Analysis of secreted Cyr61 and metalloproteinases MMP2, MMP7 and MMP9 from equal number of control or Doxy-treated cells (2 μg/ml) for 72 h. Conditioned media were used to prepare total soluble fraction of secretome by differential centrifugation as in figure 2E. After concentration by methanol/chloroform precipitation, pellet was resuspended in PBS-5mM EDTA for immunoblotting analyses (see Material and Methods). Results are representative of three independent experiments. **F.** SUM159PT-Tet-On-SrcDN cells were cultured with or without Doxy (1 μg/ml) for 72 h. Expression of SrcDN and Cyr61 was determined by immunoblotting in total cell extracts and in secretome (fraction S3, see Figure 4A). Results are representative of three independent experiments.

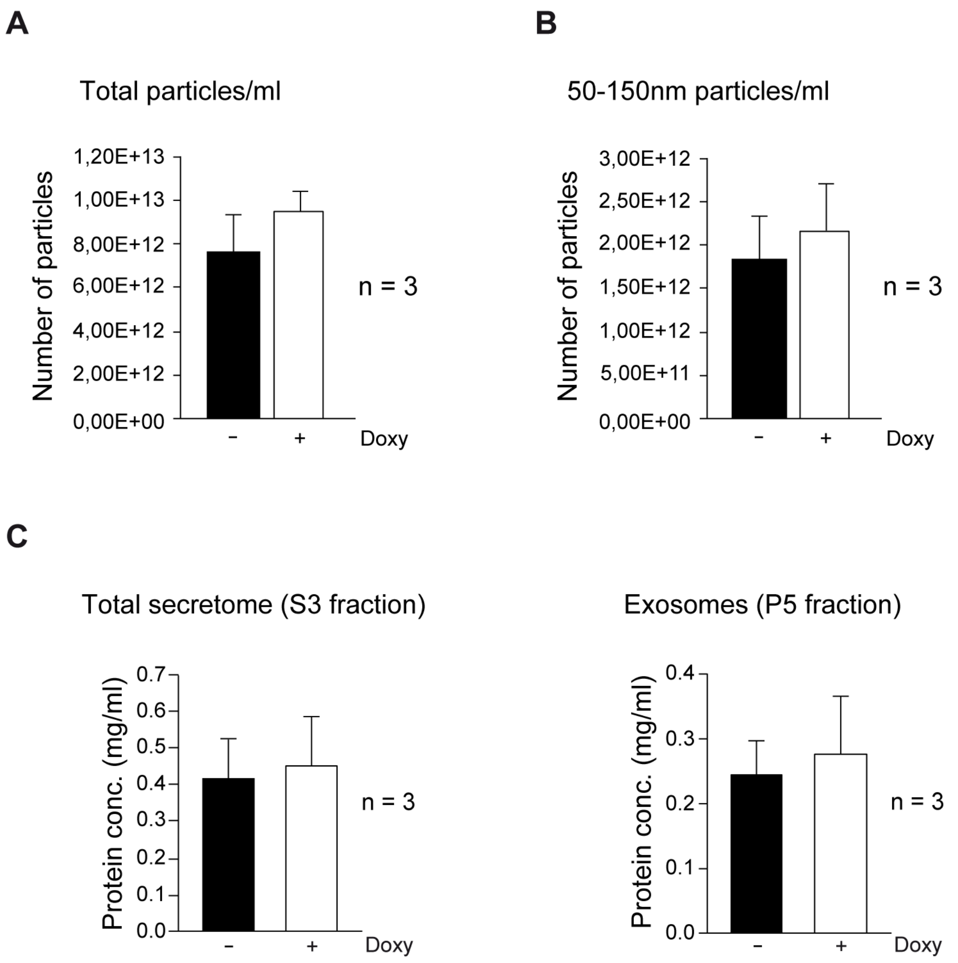

Supplementary Figure S4: Analyses of extracellular vesicles size and protein concentration upon c-Src suppression in MDA-MB-231 cells. Conditioned media from MDA-MB-231-Tet-On-shRNA-c-Src cells cultured in absence or presence of Doxy (2 µg/ml) for 72 h were collected, and the fraction P5 (Figure 4A) analyzed by Nanoparticle Tracking Analysis (NTA) with Nanosight LM10 and NTA 2.3 Software (Nanosight, Wiltshire, UK). The number and size of the extracellular vesicles were then determined. **A.** Total number of extracellular vesicles. **B.** Vesicles of a size between 50 to 150 nm that we considered contained the exosomal fraction. **C.** Total protein concentration was determined in fractions S3 and P5 of cultures grown with or without Doxy. No significant differences were observed in these analyses between untreated or Doxy-treated cells.

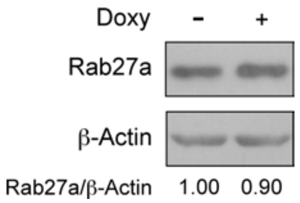

Supplementary Figure S5: Expression of Rab27a in MDA-MB-231-Tet-On-shRNA-c-Src cells. Cell extracts from untreated and Doxy-treated cells (72 h, 2 µg/ml) were used to detect by immunoblotting the levels of Rab27a and ß-actin as loading control. Results are representative of three independent experiments.

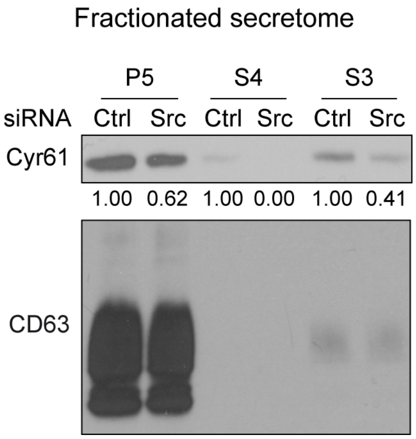

Supplementary Figure S6: Analyses of the distribution of Cyr61 and CD63 in secretome of SUM159PT transiently transfected with siRNA-c-Src. Fractionation of secretome by differential centrifugation from SUM159PT cultures transiently transfected with siRNA-c-Src. After protein concentration by methanol/chloroform precipitation of fractions, expression of Cyr61 and CD63 was analyzed by immunoblotting. ImageJ densitometry quantification expressed in arbitrary units, and considering the Ctrl as 1 for each fraction.

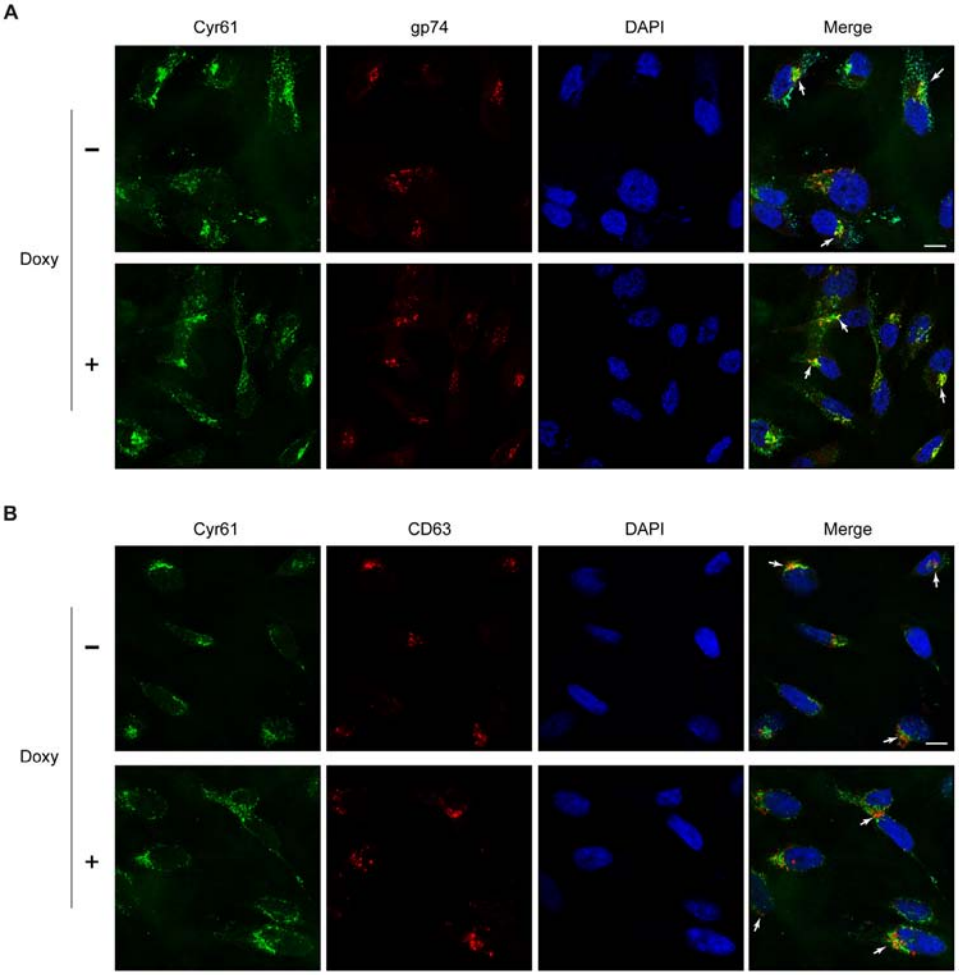

Supplementary Figure S7: Confocal analyses of Cyr61 cellular distribution. Co-localization studies of **A.** Cyr61 with gp74 (cis-Golgi marker) or **B.** CD63 (late-endosomal and exosomal marker) by scanning confocal microscopy in cells grown with or without Doxy (2 µg/ml Doxy) for 72 h (Bar = 10 µm). White arrows indicate co-localization. Pearson's coefficients for Cyr61 co-localization with gp74 and CD63 were 0.60 and 0.54 respectively, in absence of Doxy, and 0.64 and 0.51 respectively, in presence of Doxy.
